# Coenzyme Q10 Supplementation in Athletes: A Systematic Review

**DOI:** 10.3390/nu15183990

**Published:** 2023-09-15

**Authors:** Matheus Santos de Sousa Fernandes, Débora Eduarda da Silvia Fidelis, Felipe J. Aidar, Georgian Badicu, Gianpiero Greco, Stefania Cataldi, Gabriela Carvalho Jurema Santos, Raphael Frabrício de Souza, Luca Paolo Ardigò

**Affiliations:** 1 Graduate Program in Neuropsychiatry and Behavioral Sciences, Center for Medical Sciences, Federal University of Pernambuco, Recife 50740-600, Pernambuco, Brazil; theusfernandes10@hotmail.com; 2 Programa de Pós-Graduação em Biologia Aplicada à Saúde, Centro de Biociências, Universidade Federal de Pernambuco, Recife 50740-600, Pernambuco, Brazil; deeborafidelis_@hotmail.com; 3 Department of Physical Education, Federal University of Sergipe, São Cristovão 49100-000, Sergipe, Brazil; fjaidar@gmail.com (F.J.A.); raphaelctba20@hotmail.com (R.F.d.S.); 4 Department of Physical Education and Special Motricity, Faculty of Physical Education and Mountain Sports, Transilvania University of Braşov, 500068 Braşov, Romania; 5 Department of Translational Biomedicine and Neuroscience (DiBraiN), University of Study of Bari, 70124 Bari, Italy; gianpiero.greco@uniba.it (G.G.); stefania.cataldi@uniba.it (S.C.); 6 Graduate Program in Nutrition, Federal University of Pernambuco, Recife 50740-600, Pernambuco, Brazil; gaby9carvalho@gmail.com; 7 Department of Teacher Education, NLA University College, 5812 Oslo, Norway; luca.ardigo@nla.no

**Keywords:** exercise, physical activity, physical training, sports nutrition

## Abstract

Background: To summarize available evidence in the literature on the impacts of CoQ_10_ supplementation on metabolic, biochemical, and performance outcomes in athletes. Methods: Six databases, Cochrane Library (33 articles), PubMed (90 articles), Scopus (55 articles), Embase (60 articles), SPORTDiscus (1056 articles), and Science Direct (165 articles), were researched. After applying the eligibility criteria, articles were selected for peer review independently as they were identified by June 2022. The protocol for this systematic review was registered on PROSPERO (CRD42022357750). Results: Of the 1409 articles found, 16 were selected for this systematic review. After CoQ_10_ supplementation, a decrease in oxidative stress markers was observed, followed by higher antioxidant activity. On the other hand, lower levels of liver damage markers (ALT); Aspartate aminotransferase (AST); and Gamma-glutamyl transpeptidase (γGT) were identified. Finally, we found a reduction in fatigue indicators such as Creatine Kinase (CK) and an increase in anaerobic performance. Conclusions: This systematic review concludes that supplementation with orally administered CoQ_10_ (30–300 mg) was able to potentiate plasma antioxidant activity and anaerobic performance, reducing markers linked to oxidative stress and liver damage in athletes from different modalities aged 17 years old and older.

## 1. Introduction

The evidence demonstrates numerous benefits in human health promoted by the practice of Physical Activity (PA) and Physical Exercise (PE), including the reduction in the risk of chronic and cardiometabolic diseases and the risk of early mortality [1,2]. On the other hand, strenuous PE, which is generally associated with a great demand for physical effort, intensity, and duration, enhances the development of physical abilities and high performance, demanding a great energy demand from its practitioners [3]. These exhaustive practices can establish large proportions of damage to organ systems, resulting in inflammatory processes, chronic muscle injuries, pain, and proteolysis that can lead to cell apoptosis [4,5]. The safe use of supplements becomes necessary, as it is a viable and reliable way to meet high nutritional demands that cannot only be obtained from your daily diet and improve athletic performance [6,7].

The use of nutritional supplements serves different purposes around the world, but only 5% are intended for high-performance athletes to supplement food and improve metabolic function and performance [8,9]. In eukaryotic cells, Coenzyme Q10 (CoQ_10_) is present in three oxidation states: ubiquinol (Q10 H_2_), ubisemiquinone, and ubiquinone in its full oxidation state. It participates in aerobic processes to produce Adenosine Triphosphate (ATP), acting directly as an electron carrier in oxidative phosphorylation that occurs in mitochondria, as well as assisting in the maintenance of the redox cycle by assisting in the antioxidant response [10,11,12,13]. Its biosynthesis pathway occurs via the side chain of the polyisoprenoid CoQ, starting from acetyl-CoA and passing through mevalonate and isopentenyl pyrophosphate, the same as cholesterol. Studies show that CoQ_10_ supplementation promotes an increase in the levels of this substance, mainly in the mitochondrial region of various tissues such as the brain, heart, and kidneys [14,15].

In addition, they can act to combat the excess production of Reactive Oxygen Species (ROS), which are part of the pathophysiology of numerous chronic diseases, including cardiometabolic and neurodegenerative diseases [16,17]. It is known that athletes of different levels (amateur to elite) modalities can produce high levels of ROS associated with reduced antioxidant defenses, causing Oxidative Stress (OS) [18].

Some systematic reviews have demonstrated the benefits of CoQ_10_ supplementation in health and disease conditions [19,20,21]. Furthermore, Drobnic et al. (2022) observed an increase in plasma levels of CoQ_10_ after its supplementation, promoting benefits in performance indicators and recovery in athletes of different sports [22]. However, different from previous findings, in this review, we sought to identify the impacts of CoQ_10_ supplementation on outcomes related to body composition, biochemistry, and performance parameters since they are not entirely clear in athletes of different levels and modalities. Therefore, this systematic review aims to summarize available evidence in the literature on the impacts of CoQ_10_ supplementation on body composition, biochemical, and performance outcomes in athletes.

## 2. Methods

The present systematic review followed the Preferred Reporting Items for Systematic Reviews and Meta-Analysis (PRISMA) guidelines and was previously registered on PROSPERO (CRD42022357750).

### 2.1. Eligibility Criteria

Eligibility criteria were previously selected to minimize the risk of bias. The inclusion and exclusion criteria followed the PICOS (Population/Intervention/Control/Outcomes/Study) (Table 1). There were no restrictions on language or publication date. Studies that did not meet the eligibility criteria, review publications, letters, duplicates, and the presence of data used in different studies were excluded.

### 2.2. Information Sources and Search Strategy

The search strategy was carried out during the period from May to June 2022. The databases used were Cochrane Library; PubMed (Medline), Scopus, Science Direct, Embase, and SPORTDiscus. The search strategies used for Cochrane Library; PubMed (Medline), Embase; and Scopus were ((((Coenzyme Q10) OR (co-enzyme Q10)) OR (CoQ 10)) OR (Ubiquinone)) AND ((((((Athletes) OR (Athlete)) OR (Professional Athletes)) OR (Elite Athletes)) OR (College Athlete)) OR (College Athletes)); Science Direct: ((((Coenzyme Q10) OR (co-enzyme Q10)) OR (CoQ 10)) OR (Ubiquinone)) AND ((((Athletes) OR (Professional Athletes)) OR (Elite Athletes)) OR (College Athletes)); SPORTDiscus: ((((“Coenzyme Q10”) OR (“co-enzyme Q10”)) OR (“CoQ 10”)) OR (“Ubiquinone”)) AND ((((((“Athletes”) OR (“Athlete”)) OR (“Professional Athletes”)) OR (“Elite Athletes”)) OR (“College Athlete”)) OR (“College Athletes”)). Filters were also used in the databases [Humans and type of publication] (Supplementary Table S1).

### 2.3. Selection and Data Collection Process

**Figure 1:**
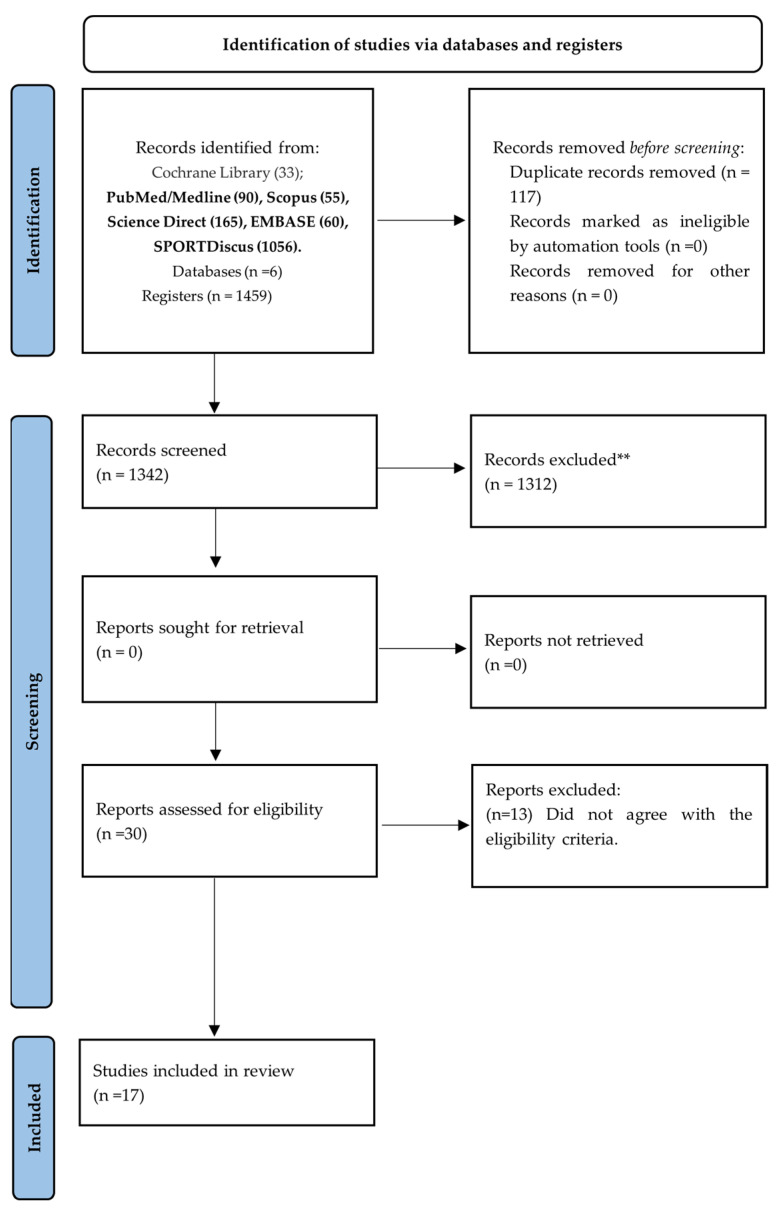
PRISMA 2020 flow diagram for new systematic reviews, which included searches of databases and registers only. Consider, if feasible to do so, reporting the number of records identified from each database or register searched (rather than the total number across all databases/registers). ** If automation tools were used, indicate how many records were excluded by a human and how many were excluded by automation tools.

The screening was performed by reading the title, abstract, and full text. The selection of studies was performed by two independent researchers (MSSF and GCJS). Data was extracted via two independent researchers. Discrepancies were resolved by a third rater (DEdSF) (Figure 1).

### 2.4. Data Items

Data were extracted about the study (Author and year); sample characteristics (age, sex, sample size); information about the type of athletes or category of athletes (amateurs, professionals, or elite); modality or type of sport practiced; and protocol CoQ_10_ supplementation (route and dose of administration). In the absence of information, data were not considered. Data were collected as follows:

(1) Body composition outcomes such as Body Mass Index; Fat percentage (%); and Body mass or Weight (kg).
(2) REDOX Balance and Oxidative Stress: Carbonyls; Catalase; Malonaldehyde (MDA); Glutathione Peroxidase (GPx); 8-ODHdG; Myeloperoxidase (MPO); NADPH oxidase; Cytosolic ROS; H_2_O_2_; Hydroperoxides; Scavenging activity against superoxide anion; TAC; TAS; Oxidative DNA damage; and Xanthine Oxidase (XO).
(3) Biochemical outcomes: Alanine aminotransferase (ALT); Aspartate aminotransferase (AST); Blood urea nitrogen; Creatinine; Creatine Kinase (CK); Creatine phosphokinase (CPK); Free Fatty Acids (FFA); Gamma-glutamyl transpeptidase (γGT); Glucose; High-Density Lipoprotein (HDL); Lactate; Lactic acid clarity; Lactate score; Lactate pyruvate ratio score; non-esterified fatty acid (NEFA); Myoglobin; Phospholipids; Total cholesterol; Total bilirubin; Triglycerides; Uric Acid; and urine creatinine.
(4) Performance outcomes were divided and shown in Table 2.

### 2.5. Methodological Quality Assessment

The “Joanna Briggs Institute (JBI) Critical Appraisal Checklist for Analytical Randomized Controlled Trial and Non-Randomized Experimental Studies” [21] was used to verify the methodological quality of the articles included. The JBI consists of eight questions that assess the methodological quality of the articles based on the following criteria: selection of participants, confounding variables, validity, and reliability of the results. The questions were answered with “Yes”, “No”, or “Undefined”. When the answer was “yes”, a score was given; when the answer was “no” or “undefined”, no score was given. The score for each article was calculated as a percentage and the quality of each study was rated as high (80–100%), fair (50–79%), or low (50%). All studies were independently reviewed by two reviewers. Discrepancies between raters were resolved by consensus.

## 3. Results

### 3.1. Characterization of Included Studies

A total of 1459 studies were identified between searches in the databases. Cochrane Library (n = 33); PubMed/Medline (n = 90); Scopus (n = 55); Science Direct (n = 165); Embase (n = 60); SPORTDiscus (n = 1056)]. After the removal of duplicates (n = 117), 1342 articles were screened for the inclusion process. Then, 1312 publications were excluded after observing the title/abstract, and the remaining 30 studies were selected for reading the full text. Finally, 17 studies were included in the present systematic review. The process of search, selection and inclusion of studies is summarized in the flow diagram of the PRISMA statement (Figure 1). The present study includes articles published between 1991 and 2020 (Table 3). The studies were performed in Iran [23,24,25], Japan [26,27,28], the United States of America (USA) [29,30], Sweden [31,32], Spain [33], United Kingdom (UK) [34], Australia [35], Brazil [36], Finland [37], and Italy [38].

Regarding gender, 14 studies used only males [23,24,25,26,27,28,29,31,32,33,35,36,37,38]; on the other hand, 2 studies were carried out with both genders [30,34]. The mean age of participants ranged from 17 to 46.3 years.

Within the studies, heterogeneity in sports was observed including cycling, running, triathlon, climbing, swimming, martial arts and fights, rugby, cross-country skiing, tennis, and ice hockey. Eight of the included studies [28,29,30,31,32,33,34,38] used only amateur athletes and eight elite athletes [23,24,25,26,27,35,36,37]. Regarding the protocol of CoQ_10_ supplementation, all studies included the use of the oral route for the administration of the supplement. There were different dosages used in the studies: 300 milligrams (mg) of CoQ_10_ (n = 5), 100 mg of coenzyme Q_10_ (n = 4), 200 mg of CoQ_10_ (n = 2), 90 mg of CoQ_10_ (n = 2), 250 mg of CoQ_10_ (n = 1), 120 mg of CoQ_10_ (n = 1), and 30 mg of coenzyme Q_10_ (n = 1). The time of CoQ_10_ administration ranged from 11 to 60 days.

### 3.2. Body Composition and Biochemical Outcomes

**Figure 2:**
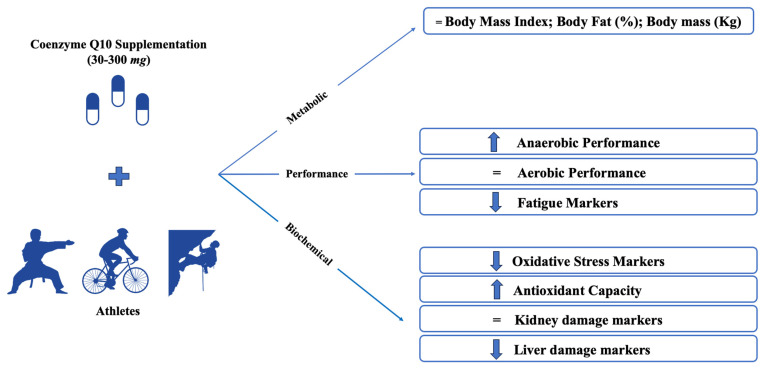
Impacts of Coenzyme Q10 supplementation on body composition, biochemical and performance outcomes of athletes from different modalities. BMI: Body Mass Index; mg: milligrams.

Body composition is described using the body mass index (BMI), body fat (%, kg), and body mass (kg) (Table 4). All protocols showed no changes in BMI, fat mass, and body mass. However, studies by Mohammadi et al., 2020 [36] and Holloway et al., 2014 [34] showed a reduction in BMI and body fat, respectively. The biochemical parameters (REDOX balance, lipid and glucose profile, kidney/liver damage markers, and bioenergetic outcomes) are shown in Figure 2. REDOX balance outcomes are evaluated by pro and antioxidant markers. CoQ_10_ supplementation causes an increase in indicators of antioxidant activity such as CAT, TAC, and TAS. Changes were not observed in the GPx. Regarding the pro-oxidant markers, there was either a reduction (Basal and induced membrane hydroperoxides, 8-OHdG, LPO, Carbonyls, MPO, XO, and Cytosolic ROS) or no change (H_2_O_2_, scavenging activity against superoxide anion, oxidative DNA damage, and hypoxanthine) of the markers. CoQ_10_ supplementation did not promote changes in FFA, NEFA, phospholipids, triglycerides, total cholesterol, and glucose levels. Only HDL levels were reduced Holloway et al., 2014 [34]. There were no changes in renal function markers (creatinine, uric acid, and blood urea nitrogen). However, liver function markers such as bilirubin, AST, ALT, and γGT decreased (Castro et al., 2012 [33]; Emani et al., 2018 [25]; Suzuki et al., 2020 [28]).

### 3.3. Fatigue Markers

Regarding fatigue markers (Table 4), no differences were observed in max lactate, sub-max lactate, the workload at the lactate threshold, time to exhaustion, and RPE. However, in the protocol by Mohammadi et al., 2020 [36], an increase in the fatigue index was observed, Snider et al., 1992 [39]. On the other hand, the percentage of fatigue was lower in the protocol used by Suzuki et al., 2020 [39]. Six studies evaluated CK levels after supplementation where five showed a reduction, while for CPK levels, no differences were observed [30]. Three studies showed no difference in lactate levels after supplementation [30] (Holloway et al., 2014 [34]; Snider et al., 1992 [39]). Only one study demonstrated a reduction after CoQ_10_ ingestion (Emani et al., 2018 [25]). Likewise, there was a reduction in myoglobin and enzyme levels of NADPH oxidase and LDH in all protocols.

### 3.4. Performance Outcomes

Evaluated performance outcomes via respiratory, hemodynamic, neuromuscular, and bioenergetic parameters (Table 5). No differences were observed in VO_2_ max and peak levels in five studies. Only in the study by Yikioski et al., 1997 [37] was there an increase in VO_2_ max associated with the AET and ANT increase. Additionally, only Deichmann et al. 2012 observed an increase in the time to anaerobic threshold after CoQ_10_ supplementation, with no differences observed in hemodynamic outcomes. Six studies included in the present review evaluated different neuromuscular variables. The supplementation protocol was able to increase the total work (W) [29], muscle strength [30], aerobic power (W), average power (W), maximum power (W), and minimum power (W), respectively [36]. However, only one study showed a significant reduction in the average of mean power output; power output (W·kg·bw); 10 × 10 s test (W·kg·bw); 15 × 10 s (W·kg·bw), [31]. Four studies did not show significant changes in neuromuscular variables [25,31,32,36].

### 3.5. Methodological Quality Assessment

All studies demonstrated fair quality (75%). The identification and control of confounders were not evaluated in all studies. However, the inclusion criteria, description of context participants, reliable and valid measurements, and adequate statistical analysis were considered (Table 6).

## 4. Discussion

This systematic review aimed to summarize the findings in the literature about the impacts of coenzyme Q10 supplementation on body composition, and biochemical and performance markers in athletes of different modalities. Within the main results, we found that the protocol did not promote changes in body composition, kidney function, and aerobic performance. However, after CoQ_10_ supplementation, there was a decrease in oxidative stress indicators, followed by an increase in antioxidant capacity. Additionally, improvement in liver function and fatigue markers was also observed, with a consequent increase in the anaerobic performance assessed by neuromuscular variables, including average of mean power; power output, power at least (W), 10 × 10 s test, 15 × 10 s, total work, muscle strength, and bioenergetic outcomes such as the time to anaerobic threshold.

Corroborating our results, Ghavami et al., 2020 [40], when performing a systematic review with meta-analysis using twenty randomized clinical trials, did not observe significant differences in anthropometric markers including weight, BMI, and waist circumference in non-athlete adults after CoQ_10_ supplementation [40]. It is understood that changes in body measurements and composition may affect the functioning of mitochondria, which play a crucial role in producing energy via cellular signaling pathways that rely on the oxidation of carbohydrates and fatty acids [41,42]. Fatty acids are transported to the interior of the mitochondria via transport proteins located in its outer membrane known as Carnitine PalmitoylTransferase I (CPT-I) and II (CPT-II), starting several reactions linked to mitochondrial b-oxidation [43]. These reactions effectively contribute to the control of lipid metabolism, preventing its accumulation, which is directly linked to metabolic disorders including overweight and obesity [44].

We observed that the CoQ_10_ supplementation increased antioxidant activity and was associated with lower levels of OS markers in athletes. Ho et al. demonstrated that 12 weeks of supplementation with 300 mg of CoQ_10_ increased the TAC of Taekwondo and soccer athletes [45]. In this sense, the use of antioxidant supplements in sports is recommended since athletes are exposed to sports with high demands of physical effort contained in training and competitions [46,47]. This scenario promotes deleterious metabolic effects including excessive production of Reactive Oxygen Species (ROS) associated with low activity of antioxidant defenses, promoting OS, and inflammation impacting health and athletic performance [48,49].

The results showed no changes in lipid profile (FFA, HDL, NEFA, phospholipids, triglycerides, and total cholesterol), glucose, and markers of kidney damage after supplementation with CoQ_10_ supplementation in athletes. Studies with this population are scarce and need to establish reliable conclusions. However, the efficiency of CoQ_10_ supplementation in non-athlete subjects and those affected by different pathologies including type 2 diabetes (T2DM) are consolidated [50].

Zahedi et al. used 150 mg of CoQ_10_ in 20 patients with T2DM for 12 weeks; after the protocol, there was a significant reduction in fasting plasma glucose, insulin, and hemoglobin A1C was identified. Furthermore, these findings suggest that the use of CoQ_10_ plays an important role in the control of carbohydrate metabolism [51]. The participation of this macronutrient is essential to produce ATP, which occurs mainly via aerobic reactions including oxidative phosphorylation, which has the help of CoQ_10_ in the connectivity of complexes I, II, and III of the electron transport chain [52]. Moreover, bioenergetic disturbances that result in a decrease in ATP intake are linked to a drop in athletic performance [53,54].

The data showed a decrease in liver damage markers after the use of CoQ_10_. Evidence in non-athletes and athletes points to impacts on liver function. Farsi et al. performed 100 mg of CoQ_10_ supplementation for 12 weeks in 41 patients with Non-Alcoholic Fatty Liver Disease (NAFLD); their results showed a decrease in AST and γGT, as well as lower levels of inflammation and degree of steatosis, which were positive changes in the prognosis clinic of these patients [55]. On the other hand, Liao et al. found no significant differences in hepatic mitochondrial functionality in adolescent Chinese athletes [56].

This systematic review concludes that CoQ_10_ supplementation can effectively reduce fatigue markers and improve anaerobic performance in athletes. However, there is no significant effect on aerobic capacity. It is important to note that voluntary muscle fatigue is influenced by various factors and is regulated by both central and peripheral mechanisms [57]. Elevated levels of fatigue are identified via biochemical markers in the blood that can effectively alter athletic performance [58]. Therefore, sports supplementation strategies should be indicated to contain the deleterious effects on the athletes. Drobnic et al. summarized evidence systematically and pointed out that CoQ_10_ supplementation can decrease muscular fatigue by promoting low levels of inflammatory response and muscle damage markers including creatine kinase and myoglobin [22]. Unlike our work, the authors included studies with non-athletes and athletes.

In aerobic capacity indicators, we did not observe improvements after CoQ_10_ supplementation, according to the body of evidence available in the present systematic review. Similarly, Liao demonstrated that 100 mg of CoQ_10_ supplementation for 28 days was not able to improve the VO_2_Max of adolescent swimmers [56]. However, we observed significant changes in components of anaerobic performance including anaerobic threshold, muscle strength (number of repetitions), muscle power, and total work measured in Watts after CoQ_10_ supplementation. High levels of these anaerobic performance variables are essential for sports performance and obtaining results in different modalities [59]. Recent findings indicate that these advantages could lead to (1) reduced OS production by boosting antioxidant capacity; (2) decreased production of cellular indicators of inflammation and muscle exhaustion; and (3) enhanced muscular endurance and a combination of aerobic and anaerobic metabolic pathways that work together to generate more energy availability needed for high-intensity and short-duration physical activities [60].

### 4.1. Limitations and Strengths

This report acknowledges some limitations in the studies included. Firstly, the amount of CoQ_10_ supplementation and duration varied among athletes, making it challenging to determine the necessary dose for optimal health and performance benefits. Secondly, different sports have varying physical, technical, and bioenergetic characteristics, so studies stratified by sport would provide more insights into the mechanisms of CoQ_10_ supplementation. Lastly, there was heterogeneity in the participants. Despite these limitations, this report is the first to examine the effects of CoQ_10_ supplementation on athletes’ body composition, REDOX balance, lipid and glycemic profiles, and markers of kidney and liver damage. The findings on fatigue markers and athletic performance were displayed in an easy-to-understand format, making it useful for future studies and prescribing to various populations of athletes and non-athletes.

### 4.2. Future Directions and Perspectives

The purpose of the present work was to systematically demonstrate that CoQ_10_ supplementation performed is capable of exerting biological benefits at the molecular and cellular levels via the promotion of control in the oxidative balance since oxidative stress is part of the pathophysiology of several diseases, which can affect athletes exposed to high demands of physical and mental effort. At the clinical level, reducing liver damage markers, which, when deregulated, point to a possible state of aggression to the liver due to multiple pathological conditions. In addition, we observed significant improvements in anaerobic performance and fatigue indicators. Finally, the use of CoQ_10_ or any supplement must respect the biological individuality of each athlete and the specificity of their modality, as well as be prescribed responsibly by a legally qualified professional. We recommend that further studies indicate that safe CoQ_10_ supplementation can be used to promote a high level of athletic performance in various modalities. In addition, they demonstrate their relevance for maintaining health and, mainly, as an adjunct aid to the treatment and rehabilitation of chronic degenerative diseases, including cardiovascular, metabolic, and aging-related diseases.

## 5. Conclusions

This systematic review concludes that supplementation with CoQ_10_ (30–300 mg) orally administered was able to potentiate antioxidant activity and anaerobic performance, reducing markers linked to oxidative stress and liver damage in athletes from different modalities aged 17 years old and older.

## Figures and Tables

**Table 1:** PICOS strategy.

	Inclusion Criteria	Exclusion Criteria
Population	Athletes from 17 years old	Non-athletes
Intervention	Coenzyme Q10 supplementation	No Coenzyme Q10 supplementation or presence of another type of supplementation or medication
Control	Subjects who did not receive COQ_10_ supplementation from 17 years of age	Patients with diseases, undergoing medication, or exposed to pharmacological interventions
Outcomes	Metabolic, physiological, and athletic performance parameters	No Metabolic, physiological, and athletic performance parameters
Study	Intervention	Reviews; Case reports; Letters to editors; comments, etc.

**Table 2:** Variables, conceptual description, and performance indicators in athletes after CoQ_10_ supplementation.

Performance Outcomes	Description	Data Extracted from the Main Indicators
Aerobic Capacity	The ability of the body to produce energy via metabolic processes dependent on oxygen and are used to oxidize macromolecules to generate energy.	VO_2_ Máx, RER, Speed, Maximal O_2_ consumption; O_2_ uptake.
Hemodynamic profile	Refers to the description of the characteristics and behavior of an individual’s cardiovascular system.	HR, DBP, SBP, BP, Submax Pulse, and HR rate at lactate threshold.
Neuromuscular	Relationship between the nervous system and the muscles of the body to provide movement.	Total Work, Muscle strength, Power, 10 × 10-s, 15 × 10-s, 30-s tests, Maximal workload.
Anaerobic threshold parameters	Related to the point during physical exertion when lactic acid production begins to exceed the body’s ability to remove it, resulting in a significant increase in blood.	ANT, AET, Workload at lactate threshold

Notes: AET: Aerobic threshold; ANT: Anaerobic threshold; VO_2_ Máx: Maximum volume of oxygen; BP: Blood Pressure, DBP: Diastolic Blood Pressure; HR: Heart Rate.

**Table 3:** Sample, sports modalities, and protocol of CoQ_10_ supplementation characteristics.

Author, Year	Age (Yrs)	Gender	Modality	Country	*n*	Category	Protocol of CoQ_10_ Supplementation		
							Route of Administration	Dosage (mg)	Administration Time
Braun et al., 1991 [29]	21.9 *	M	Cyclists	USA	12	Amateurs	OA	100	60 days
Castro et al., 2012 [33]	41.2	M	Runner	Spain	10	Amateurs	OA	30	Uninformed
Deichmann et al., 2012 [30]	63.6	M/F	Triathlon	USA	19	Amateurs	OA	200	6 weeks
Emani et al., 2018 [24]	17.0	M	Swimmers	Iran	36	Elite	OA	300	2 weeks
Emani et al., 2018 [25]	17.0	M	Swimmers	Iran	36	Elite	OA	300	2 weeks
Emani et al., 2020 [23]	17.0	M	Swimmers	Iran	36	Elite	OA	300	2 weeks
Holloway et al., 2014 [34]	46.3	M/F	Climbers	UK	23	Amateurs	OA	300	22 days
Kon et al., 2008 [26]	20.5	M	Kendo	Japan	18	Elite	OA	100	2 weeks
Malm et al., 1997 [31]	20–34	M	Runner and Cyclists	Sweden	18	Amateurs	OA	120	22 days
Mohammadi et al., 2020 [36]	18.5	M	Wrestlers	Brazil	20	Elite	OA	100	6 weeks
Orlando et al., 2018 [38]	26.0	M	Rugby	Italy	21	Amateurs	OA	200	4 weeks
Ostman et al., 2012 [32]	19–44	M	Runner, Cross-country skiers, tennis, ice hockey	Sweden	23	Amateurs	OA	90	8 weeks
Shimizu et al., 2015 [27]	20.4	M	Kendo	Japan	18	Elite	OA	300	2 weeks
Suzuki et al., 2020 [28]	18–25	M	Runner	Japan	16	Amateurs	OA	100	11 days
Weston et al., 1997 [35]	24.8	M	Cyclists and Triatlon	Australia	18	Elite	OA	250	4 weeks
Yikioski et al., 1997 [37]	-	M	Cross-country Skiers	Finland	25	Elite	OA	90	12 weeks

Notes: F: Female; M: Male; mg: milligrams; *n*: number of participants; OA: Oral Administration; Yrs: years. * Mean of age.

**Table 4:** Body composition and biochemical outcomes of athletes after CoQ_10_ supplementation.

Author, Year	Body Composition	Biochemical Parameters			
		REDOX Balance	Lipid and Glucose Profile	Kidney/Liver Damage Markers	Fatigue Markers
Braun et al., 1991 [29]	-	= MDA	-	-	-
Castro et al., 2012 [33]	-	↑ CAT; TAS ↓ Basal and induced membrane hydroperoxides and 8-OHdG = GPx	= Phospholipids; TG; total cholesterol	= Urine creatinine ↓ Total bilirubin	-
Deichmann et al., 2012 [30]	-	-	-	-	= CPK, LA score; LA pyruvate ratio score
Emani et al., 2018 [24]	= BMI, BF (%); Body mass (kg);	↓ LPO; ↑ TAC	-	-	↓ CK; Myoglobin
Emani et al., 2018 [25]	= BMI, BF (%); Body mass (kg);	↓ Carbonyls; 8-OhdG = H_2_O_2_	-	↓ ALT; AST; GGT	↓ CK, LA, NADPH oxidase
Emani et al., 2020 [23]	= BMI, BF (%); Body mass (kg);	↓ MPO; XO	-	-	-
Holloway et al., 2014 [34]	= Body mass (kg); ↓ BMI, BF (kg)	-	↓ HDL; Total cholesterol = Glucose; TG = NEFA	= Creatinine	= LA
Kon et al., 2008 [26]	= Body weight (kg); BF (%)	↓ LPO = Scavenging activity against superoxide anion	-	-	↓ CK; Myoglobin
Malm et al., 1997 [31]	= Body weight (kg)	-	-	-	= Max lactate; Submax lactate; RPE
Mohammadi et al., 2020 [36]	= BMI; Body mass (kg)	-	-	-	↑ Fatigue index
Orlando et al., 2018 [38]	-	↓ Cytosolic ROS = Oxidative DNA damage	-	-	↓ CK; Myoglobin
Ostman et al., 2012 [32]	= BMI; Body mass (kg)	= Hypoxanthine	-	= Uric Acid	= CK
Shimizu et al., 2015 [27]	= BMI, BF (%); Body mass (kg);	-	-	-	-
Snider et al., 1992 [39]	-	-	= Glucose; FFA	-	= LA; = Time to exhaustion; RPE
Suzuki et al., 2020 [28]	-	-	-	↓ ALT; AST = Blood urea nitrogen; Creatinine; Uric Acid	↓ CK; Fatigue (%); LDH;
Weston et al., 1997 [35]	= Body mass (kg)	-	-	-	= Exhaustion
Yikioski et al., 1997 [37]	-	-	-	-	= Lactic acid clearance

Notes: ALT: Alanine aminotransferase; AST: Aspartate aminotransferase; BMI: Body Mass Index; BF: Body Fat Percentage (%); CAT: Catalase; CK: Creatine Kinase; CPK: Creatine phosphokinase; DNA: Deoxyribonucleic acid; FFA: Free Fatty Acid; GGT: Gammaglutamyltransferase; GPx: Glutathione Peroxidase; H_2_O_2_; Hydrogen peroxide; HDL: High Density Lipoprotein; LA: Lactate; LDH: Lactate Dehydrogenase; LPO: Lipoperoxidation MDA: Malondialdehyde; MPO: Myeloperoxidase; NADPH: Nicotinamide Adenine Dinucleotide Phosphate; ROS: Reactive Oxygen Species; TAS: Total Antioxidant Status; TG: Triglycerides; 8-OhdG: 8-Hydroxy-2′-deoxyguanosine; XO: Xanthine Oxidase, ↑ significant increase; ↓ significant decrease; = no significant difference.

**Table 5:** Performance outcomes of athletes after CoQ_10_ supplementation.

Author, Year	Performance Outcomes			
	Aerobic Capacity	Hemodynamic Profile	Neuromuscular Outcomes	Bioenergetic Outcomes
Braun et al., 1991 [29]	↑ aVO_2_ Máx = VO_2_; RER = RER	= HR	↑ Total Work (W)	-
Deichmann et al., 2012 [30]	= VO_2_ Máx; RER	-	↑ Muscle strength (repetitions)	= Difference in ANT; ↑ Time to anaerobic threshold
Emani et al., 2018b [25]	= VO_2_ Máx	-	= Max power (W)	-
Holloway et al., 2014 [34]	-	= HR; DBP; SBP	-	-
Malm et al., 1997 [31]	= VO_2_; Cycling VO_2_ peak; Running VO_2_ Máx; Submax VO_2_	= Submax pulse; and respiratory quotient	= 30-s test (W·kg·bw) ↓ Average of mean power output; Power output (W·kg·bw); 10 × 10 s test (W·kg·bw); 15 × 10 s (W·kg·bw)	-
Mohammadi et al., 2020 [36]	-	-	↑ Average power (W); Maximum power (W); Power at least (W) = Curl up; Press up	-
Orlando et al., 2018 [38]	= Average speed (km/h); Max speed (%); Time 75% max speed	-	-	-
Ostman et al., 2012 [32]	= Maximal O_2_ consumption (L/min); O_2_ consumption	= HR rate at lactate threshold; Maximal HR (beats/min)	= Maximal workload (W); Mean power output (W)	= Workload at lactate threshold
Snider et al., 1992 [39]	-	-	-	-
Suzuki et al., 2020 [28]	-	-	-	-
Weston et al., 1997 [35]	= O_2_ uptake; VO_2_ peak	= BP; HR	-	-
Yikioski et al., 1997 [37]	= VO_2_ Max	-	-	↑ AET; ANT;

Notes: AET: Aerobic threshold; ANT: Anaerobic threshold; aVO_2_ Máx: Average of the maximum volume of oxygen; BP: Blood Pressure; BW: Body Weight; DBP: Diastolic Blood Pressure; HR: Heart Rate; kg: Kilograms; km/h: Kilometers per hour; L/min: Liters/minutes; RER: Respiratory Exchange Ratio; Sec: Seconds; SBP: Systolic Blood Pressure VO_2_: Volume of Oxygen; VO_2_ Máx: Maximum volume of oxygen; W: Watt. ↑ significant increase; ↓ significant decrease; = no significant difference.

**Table 6:** Study quality assessment—Joanna Briggs Institute.

Studies	Q1	Q2	Q3	Q4	Q5	Q6	Q7	Q8	%
Braun, 1991 [29]	Y	Y	Y	Y	N	N	Y	Y	75
Castro, 2012 [33]	Y	Y	Y	Y	N	N	Y	Y	75
Deichmann, 2012 [30]	Y	Y	Y	Y	N	N	Y	Y	75
Emami, 2018a [24]	Y	Y	Y	Y	N	N	Y	Y	75
Emami, 2018b [25]	Y	Y	Y	Y	N	N	Y	Y	75
Emami, 2020 [23]	Y	Y	Y	Y	N	N	Y	Y	75
Holloway, 2014 [34]	Y	Y	Y	Y	N	N	Y	Y	75
Kon, 2008 [26]	Y	Y	Y	Y	N	N	Y	Y	75
Malm, 1997 [31]	Y	Y	Y	Y	N	N	Y	Y	75
Mohammadi, 2020 [36]	Y	Y	Y	Y	N	N	Y	Y	75
Orlando, 2018 [38]	Y	Y	Y	Y	N	N	Y	Y	75
Ostman, 2012 [32]	Y	Y	Y	Y	N	N	Y	Y	75
Shimizu, 2015 [27]	Y	Y	Y	Y	N	N	Y	Y	75
Snider, 1992 [39]	Y	Y	Y	Y	N	N	Y	Y	75
Suzuki, 2006 [28]	Y	Y	Y	Y	N	N	Y	Y	75
Weston, 1997 [35]	Y	Y	Y	Y	N	N	Y	Y	75
Yikioski et al., 1997 [37]	Y	Y	Y	Y	N	N	Y	Y	75

Notes: Y—YES, N—No. Q1: Was the inclusion criteria well defined? Q2: Have participants and context been described in detail? Q3: Were the measurements collected in a valid and reliable way? Q4: Were standardized and objective inclusion criteria used? Q5 Were any confounding variables found? Q6: Were strategies used to deal with confounding variables? Q7: Were the results measured validly and reliably? Q8: Was the statistical analysis used adequate?

## Data Availability

The datasets are available from Matheus Santos de Sousa Fernandes upon reasonable request.

## Supplementary Materials

The following supporting information can be downloaded at https://www.mdpi.com/article/10.3390/nu15183990/s1. Table S1: Databases and search strategies were used in this systematic review.
