# Supplementary material for: Coenzyme Q10 Supplementation in Athletes: A Systematic Review
Source: Nutrients. 2023 Sep 15;15(18):3990. doi: 10.3390/nu15183990 (PMC10535924; doi:10.3390/nu15183990)
Supplement: Supplementary file 1 [file nutrients-15-03990-s001.zip › nutrients-2593007-supplementary.pdf]

nutrients MDPI

Table S1. Databases and search strategies were used in this systematic review.

Database	Search Strategy
Cochrane Library; PubMed (Medline), Embase and Scopus	((((Coenzyme Q10) OR (co-enzyme Q10)) OR (CoQ 10)) OR (Ubiquinone)) AND ((((((Athletes) OR (Athlete)) OR (Professional Athletes)) OR (Elite Athletes)) OR (College Athlete)) OR (College Athletes))
Science Direct	((((Coenzyme Q10) OR (co-enzyme Q10)) OR (CoQ 10)) OR (Ubiquinone)) AND ((((Athletes) OR (Professional Athletes)) OR (Elite Athletes)) OR (College Athletes))
SPORTDiscus	(((("Coenzyme Q10") OR ("co-enzyme Q10")) OR ("CoQ 10")) OR ("Ubiquinone")) AND (((((("Athletes") OR ("Athlete")) OR ("Professional Athletes")) OR ("Elite Athletes")) OR ("College Athlete")) OR ("College Athletes"))
